# Patterns of Court Monitoring of Guardianship Practice in Iowa

**DOI:** 10.1093/geroni/igaa057.341

**Published:** 2020-12-16

**Authors:** Tami Swenson

**Affiliations:** Des Moines University, Des Moines, Iowa, United States

## Abstract

Guardianship, as a best practice, should provide persons with medical incapacity support for better medical care decision making, but the examination of health outcomes for those under guardianship has a dearth of evidence in practice and within the research literature. This study addresses this gap by using the publicly-available Iowa Courts online database to collect data elements useful for the investigation of the process of guardianship practice. A systematic random sample using the 99 counties within Iowa as strata was extracted for all open guardianship cases. Most of the sampled cases (96%) had private appointed guardians. Approximately 28% had case histories over 10 years, 26% 5 to 10 years, 12% 3 to 5 years, 22% 1 to 2 years, and 12% were open cases for fewer than 12 months. Court monitoring of medical conditions varied with annual, biannual, triennial, or 5 year requirements. Delinquencies in filing required medical condition documentation was common and over half of all cases had at least one delinquency in their case history and 33% had a filing delinquency within the past 2 years. Local judicial courts vary in the amount of supervision they provide guardians, and the findings from this research provide needed research to inform guardianship practices as demand increases for these services with the demographic imperative of an aging population and increasing prevalence of dementia-related health conditions.

